# Synthesis of SMA by RAFT polymerization and its dispersion of TiO_2_ in aqueous solution

**DOI:** 10.1039/d3ra00637a

**Published:** 2023-03-07

**Authors:** Deshu Cheng, Lei Zhao, Guoxiang Guan, Huaidong Lai, Juxiang Luo

**Affiliations:** a School of Resourses and Chemical Engineering, Sanming University Sanming 365004 China luojuxiang01@126.com; b College of Chemical Engineering, Fuzhou University Fuzhou 350108 China; c College of Environment and Safety Engineering, Fuzhou University Fuzhou 350108 China; d Sanming Institute of Fluorochemmical Industry Sanming 365004 China

## Abstract

Styrene-maleic anhydride copolymer (SMA) with controlled molecular weight (*M*_n_) and narrow dispersity was prepared by RAFT polymerization. The effect of reaction time on monomer conversion was investigated, and the conversion of monomer could achieve 99.1% after 24 h at the temperature of 55 °C. The synthesized SMA was characterized by Fourier transform infrared spectroscopy (FTIR), nuclear magnetic resonance (NMR) and size exclusion chromatography (SEC). The result demonstrated that the polymerization of SMA was well controlled and the dispersity (*Đ*) of SMA was lower than 1.20. Furthermore, SMA copolymers with narrow dispersity and well-regulated *M*_n_ (denoted SMA1500, SMA3000, SMA5000, SMA8000, and SMA15800, respectively) were obtained by adjusting the molar ratio of monomer to the chain transfer agent. Moreover, the synthesized SMA was hydrolyzed in NaOH aqueous solution. Then the dispersion of TiO_2_ in aqueous solution by the hydrolyzed SMA and SZ40005 (the industrial product) were studied. The agglomerate size, the viscosity and the fluidity of TiO_2_ slurry were tested. The results show that the performance of dispersity for TiO_2_ in water by SMA prepared *via* RAFT was better than that of SZ40005. It was found that the viscosity of the TiO_2_ slurry dispersed by SMA5000 was the lowest among the SMA copolymers tested, and the viscosity value of the TiO_2_ slurry with a pigment loading of 75% was only 76.6 cp.

## Abstract 2

SMA copolymers with controllable molecular weight (*M_n_*) and narrower dispersity (*Ð*) were prepared by RAFT polymerization, and the dispersing ability of SMA copolymers with different *M_n_* and *Ð* for dispersion of TiO_2_ in aqueous solution was studied.

## Graphical abstract

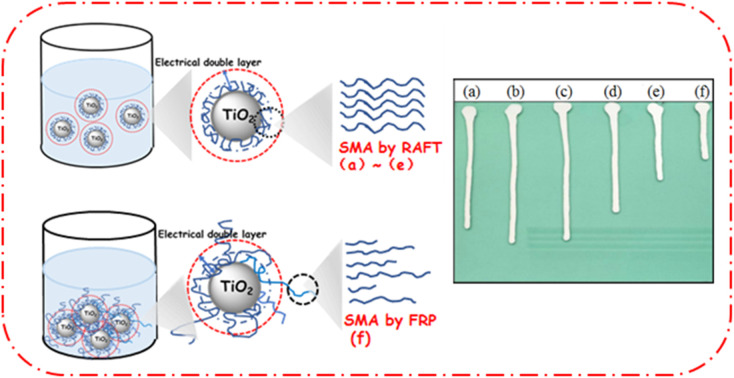

## 1 Introduction

TiO_2_ is an efficient pigment material that has been extensively used in coatings,^1,2^ papermaking,^3,4^ ink,^5,6^ plastics^7,8^ and other industries.^9^ The properties of TiO_2_ play an important role in its application, especially the dispersion ability of TiO_2_. When it is used in a coating, if the dispersion of TiO_2_ was uneven, the high whiteness, high cover and other properties of painting could not be fully exhibited. Furthermore, poor dispersing properties of TiO_2_ will affect the coating appearance and other properties of coating, such as adhesiveness, luster, rigidity, and so on. Thus, the dispersion of TiO_2_ with dispersants has attracted a wide range of interest in both theoretical studies and practical applications. Boisvert^10^*et al.* reported that the dispersion of alumina-coated TiO_2_ particles by adsorption of the sodium salt of poly(acrylic) acid (PANa) was studied. And the result suggested that both the apparent viscosity and initial yield stress of the particles decreased after adsorption of PANa. Lerch^11^*et al.* prepared a series of non-ionic ABC triblock copolymers by sequential anionic polymerizations. The non-ionic ABC triblock copolymers were used to disperse TiO_2_, showing good dispersion stability for the TiO_2_ pigments in both aqueous and non-aqueous medium. Dário^12^*et al.* investigated the stabilization of commercial TiO_2_ particles by two bulky surfactants, namely, tristyrylphenol ethoxylate (TSEO, uncharged) and tristyrylphenol ethoxylate phosphate (TSEO-P, negatively charged). And it was demonstrated that the tristyrylphenol based surfactants acted as efficient dispersants for the TiO_2_ particles in dilute and concentrated dispersions.

As a well-known copolymer with excellent performance, SMA is widely applied in emulsifier,^13^ dispersants,^14^ solubilizer,^15^ and so on. The polymer monomers of SMA are cheap and easy to polymerize. In addition, the polymer chain of SMA has many hydrophobic benzene rings and hydrophilic anhydride groups, which can form π–π conjugation with pigments to provide the particles with steric hindrance or with electric double layer to produce a barrier so as to prevent coagulation. Therefore, the modified SMA are often used as aqueous polymer dispersants.^16,17^ In order to increase the dispersibility of SMA, many methods for modification of SMA were employed, such as the amidation of anhydride group,^18,19^ the esterification of anhydride group,^20,21^ and the ionization of anhydride group^22,23^ in SMA. The current research results indicate that the dispersion property of SMA is significantly affected by the composition^22^ and the side-chain size of SMA.^24^ Nevertheless, the effects of SMA's inherent molecular structure, such as *M*_n_ or *Đ* on its dispersion property have not been reported in the literature.

SMA with well-regulated *M*_n_ could be synthesized by reversible inactivated radical polymerization (RDRP).^25^ For example, Chernikova^26^*et al.* presented that controlled free-radical copolymerization of styrene (St) and maleic anhydride (MAh) was performed in 1,4-dioxane or tetrahydrofurane solution at 60 °C using the RAFT technique. Monteiro^27^*et al.* reported that the copolymerization of St and MAh was carried out by RAFT, and the product had a low polydispersity and a predetermined molar mass. Besides, Lorigan^28^*et al.* adopted RAFT polymerization to synthesize SMA copolymers, and the SMA copolymers could be made with different molar ratios. Our research group^29^ has recently demonstrated that the controllable preparation of SMA was realized by adding vitamin C to the RAFT polymerization system of St and MAh at room temperature.

Herein, SMA with controllable structure was prepared by RAFT polymerization with *S*-1-dodecyl-*S*′-(α,α′-dimethyl-α′′-acetic acid)trithiocarbonate (DDMAT) as chain transfer agent, and the SMA aqueous solution is obtained by hydrolysis under alkaline condition. Subsequently, the dispersion of TiO_2_ in aqueous solution by the hydrolyzed SMA and the industrial product SZ40005 were carried out. Finally, the correlation between the *M*_n_ or *Đ* of SMA and their dispersion performance on TiO_2_ in aqueous solution were examined.

## 2 Experimental section

### 2.1 Materials

MAh was recrystallized from chloroform. St was passed through a basic alumina column. Azodiisobutyronitrile (AIBN) was recrystallized from ethanol. DDMAT was synthesized according to the literature.^30^ TiO_2_ (R-996) was purchased from Sichuan longmang Titanium Industry Co, Ltd. SZ40005 was purchased from Polyscope company in the Netherlands. Athanol and other reagents were of analytical grade and used as received.

### 2.2 RAFT polymerization of SMA

MAh (0.50 g, 5.1 mmol), St (0.53 g, 5.1 mmol), AIBN (6.2 mg, 3.7 × 10^−2^ mmol), DDMAT (28 mg, 0.37 mmol) and acetone (1 g) were introduced into a round-bottom flask (5 mL) equipped with a magnetic stirring, and the flask was sealed with a rubber septum. The solution was degassed by three freeze–thaw–vacuum cycles before being placed in an oil bath. Then the copolymerization was carried out at 55 °C for a prescribed time. Finally, the SMA was dissolved in acetone and precipitated in methanol prior characterization.

### 2.3 Synthesis of SMA with different *M*_n_

MAh (5.00 g, 51 mmol), St (5.30 g, 51 mmol), AIBN (7.3 × 10^−4^ mol, 3.4 × 10^−4^ mol, 2 × 10^−4^ mol, 1.3 × 10^−4^ mol, and 6.4 × 10^−4^ mol, respectively), DDMAT (7.3 × 10^−3^ mol, 3.4 × 10^−3^ mol, 2 × 10^−3^ mol, 1.3 × 10^−3^ mol, and 6.4 × 10^−3^ mol, respectively) and acetone (10 g) were introduced into a round-bottom flask (50 mL) equipped with a magnetic stirring, and the flask was sealed with a rubber septum. After 24 h reaction the samples were taken from polymerization medium for SEC experiment.

### 2.4 Hydrolysis of SMA

SMA (5.00 g), NaOH (2 g), and H_2_O (9.3 g) were added into a round-bottom flask (25 mL). The flask with a magnetic stirring was sealed with a rubber septum. The hydrolyzed SMA solution was prepared in a thermostated oil bath set at 80 °C and kept for 12 h. Then a few drops of 10% of HCl were added to the solution until the pH of solution becomes less than 3. The copolymers were collected and dried in a vacuum oven at 40 °C for 24 h.

### 2.5 Characterization of SMA

The monomer conversion was determined gravimetrically. ^1^H spectra of SMA was carried on a 400 MHz NMR (Bruker, Germany) instrument using deuterated dimethyl sulfoxide as solvent and tetramethylsilane as the internal reference. The *M*_n_ and *Đ* of SMA were determined by SEC (Waters 1515, Waters, USA) using the eluent of tetrahydrofuran in flow rate of 1.0 mL min^−1^ at 40 °C. The functional groups of the SMA and the hydrolyzed SMA were tested by FTIR (NEXUS-470, Nicolet, USA) in the range of 500 to 4000 cm^−1^.

### 2.6 Dispersion test of TiO_2_

The abovementioned hydrolyzed SMA solution (8.00 g) was adjusted to pH 9 with 40% of NaOH solution. Then propylene glycol (2.00 g) and TiO_2_ (30.0 g) were added to the solution. Subsequently, glass beads (40.0 g) and water were placed in a container. The overall solution was shocked in the shaker for 1 h. And the TiO_2_ slurry was obtained by filtering with 100 mesh iron mesh.

The test of particle size of TiO_2_ slurry was conducted using a Zetasizer Nano ZS 90(Malvern, UK). The viscosity of TiO_2_ slurry was tested by viscometer (NDJ-9S, Yarong, China) with rotor 1 at 25 °C. Tested the flow property of TiO_2_ slurry was as follows: take a glass plate, and number the sample dropping sites in turn on the plate. The TiO_2_ was dispersed by the hydrolyzed SMA with different *M*_n_ and SZ40005. Take 0.1 g of the above samples and drop them on the glass plate, respectively. After all samples have been added, slide the samples down the glass plate at the same height and the same time, then record the experiment phenomena by photographing.

## 3 Results and discussion

### 3.1 Synthesis of SMA by RAFT polymerization

The polymerization of St (an electron donor monomer) with MAh (an electron acceptor monomer) in solution at the low temperature (<80 °C) is apt to form alternating copolymer.^31,32^ The effect of reaction time on the conversion of monomer was investigated when the theoretical *M*_n_ of SMA was 6400, the molar ratio of St to MAh was 1 : 1, and the reaction temperature was 55 °C. As observed from Table 1, with the increment of the time, the monomer conversion and *M*_n_ increased gradually. When the reaction time increased from 4 h to 24 h, the monomer conversion increased from 29.5% to 99.1%, and the *M*_n_ increased from 1900 to 6300. Meanwhile, the *Đ* of polymers were less than 1.20, which showed that the RAFT copolymerization of St and MAh proceed with an apparent “living” character. After reaction of 24 h, the monomer conversion and *M*_n_ did not increase obviously with the increasing of reaction time. It may be due to there were a few unreacted monomers in the system. So in the subsequent polymerization experiments, the designed polymerization time was set at 24 h.

**Table 1:** Effect of the reaction time on RAFT polymerization of St and MAh

Time (h)	P(St-alt-MAh)_*n*_	Conversion (%)	*M*_n_	*M*_w_	*Đ*
4	*n* = 9	29.5	1900	2300	1.17
8	*n* = 12	36.8	2400	2800	1.19
12	*n* = 16	49.6	3200	3800	1.19
16	*n* = 20	65.2	4100	4800	1.15
20	*n* = 26	81.5	5200	5900	1.14
24	*n* = 31	99.1	6300	7200	1.13
30	*n* = 32	99.2	6400	7500	1.18
36	*n* = 32	99.1	6300	7200	1.13

**Fig. 1:**
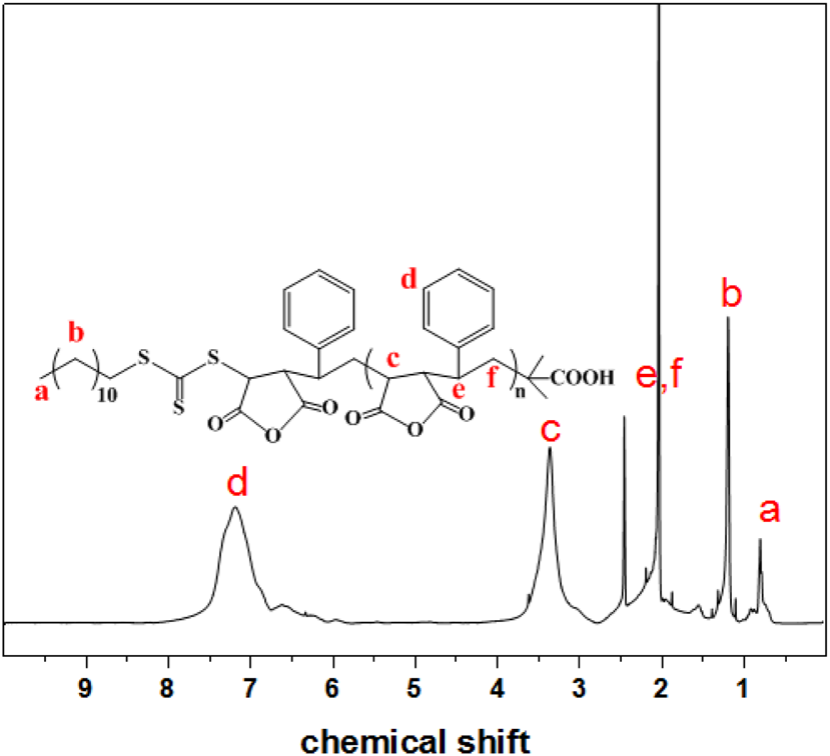
^1^H NMR spectrum of SMA (*M*_n_ = 5200).

Fig. 1 shows the ^1^H NMR spectrum of the SMA (*M*_n_ = 5200) prepared by RAFT copolymerization. The signal between 6.0–7.6 ppm (Fig. 1d) corresponds to the aromatic protons of St. The peak between 2.8–3.7 ppm (Fig. 1c) is ascribed to the methine proton of MAh unit. The peak between 1.4–2.3 ppm (Fig. 1e and f) is characteristic for the methine proton of St unit. The peaks at 0.8 and 1.2 ppm (Fig. 1a and b) are due to methyl group from the DDMAT in the polymer chain which indicate that the polymerization of St and MAh has the characteristics of “living” polymerization.

### 3.2 Synthesis of SMA with different *M*_n_

**Fig. 2:**
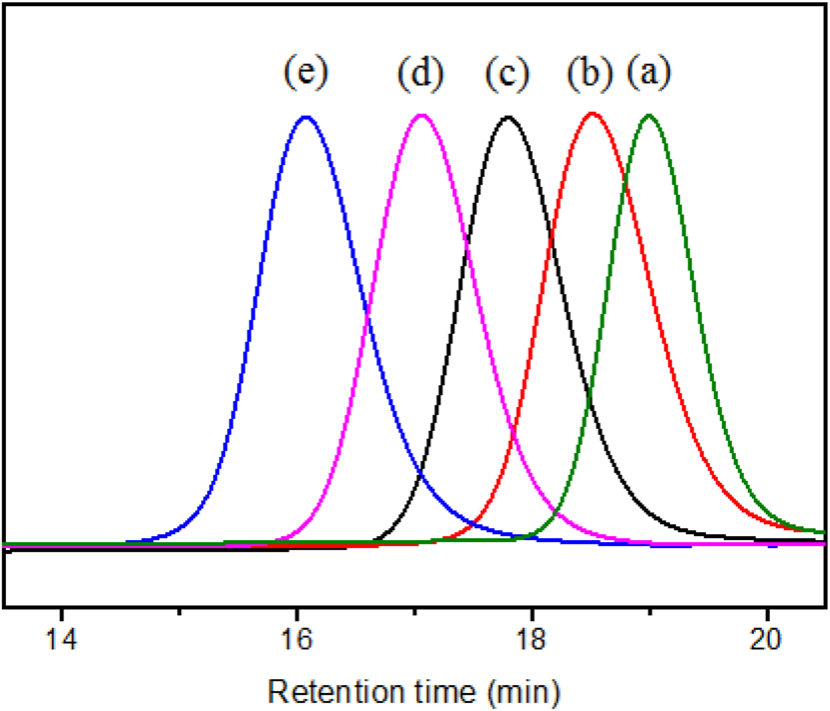
SEC spectrums for SMA with different *M*_n_, (a) SMA1500, (b) SMA3000, (c) SMA5000, (d) SMA8000, and (e) SMA15800.

In order to study the effect of SMA with different *M*_n_ on the dispersion of TiO_2_ in aqueous solution, the SMA copolymers with theoretical *M*_n_ of 1500, 3000, 5000, 8000 and 16 000 were designed. When the copolymerizations were conducted at 55 °C at the molar ratio of [St] : [MAh] = 1 : 1 with different amount of DDMAT and AIBN, SMA with different *M*_n_ were obtained. The SEC evolutions of SMA with different *M*_n_ are displayed in Fig. 2. The SEC traces are monomodal distribution, indicating the well controlled copolymerization process. And the SEC tested shows the *M*_n_ and *Đ* of SMA. That is *M*_n_ = 1500, *Đ* = 1.13 (Fig. 2a), *M*_n_ = 3000, *Đ* = 1.11 (Fig. 2b), *M*_n_ = 5000, *Đ* = 1.09 (Fig. 2c), *M*_n_ = 8000, *Đ* = 1.08 (Fig. 2d), *M*_n_ = 15 800, *Đ* = 1.15 (Fig. 2e). So the samples were recorded as SMA1500, SMA3000, SMA5000, SMA8000, and SMA15800, respectively.

**Fig. 3:**
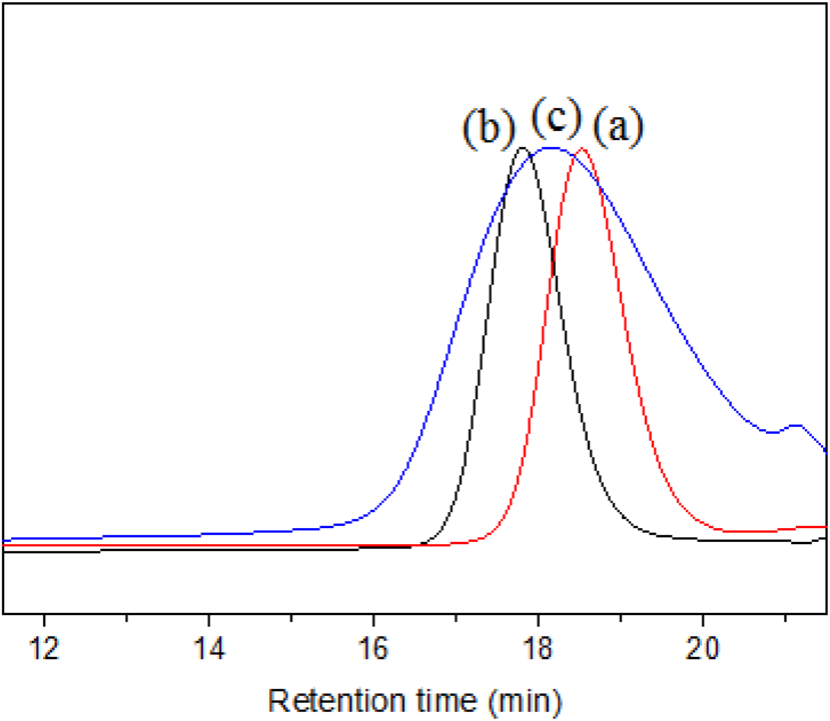
SEC spectrums for SMA3000 (a), SMA5000 (b) and SZ40005 (c).

SZ40005 is a commercial product of SMA with high acid value and low *M*_n_ from Polyscope company, which is recommended as an aqueous dispersant. Fig. 3 shows the *M*_n_ and *Đ* of SMA3000, SMA5000 and SZ40005 tested by SEC. The analytical result by SEC indicates that the *M*_n_ of SZ40005 is 2800, and the *Đ* is 1.60. The *M*_n_ of SZ40005 (Fig. 3c) is lower than SMA3000 (Fig. 3a), but it can be concluded from the plot that the high *M*_n_ macromolecules of SZ40005 are higher than that of SMA5000 (Fig. 3b). It has also been observed that the *Đ* of SZ40005 is larger than SMA3000 (*Đ* = 1.11) and SMA 5000 (*Đ* = 1.09). The *Đ* value of SZ40005 is larger than that for a typical RDRP (*Đ* < 1.50), because SZ40005 was prepared by a conventional free radical polymerization.

### 3.3 Hydrolysis of SMA

**Fig. 4:**
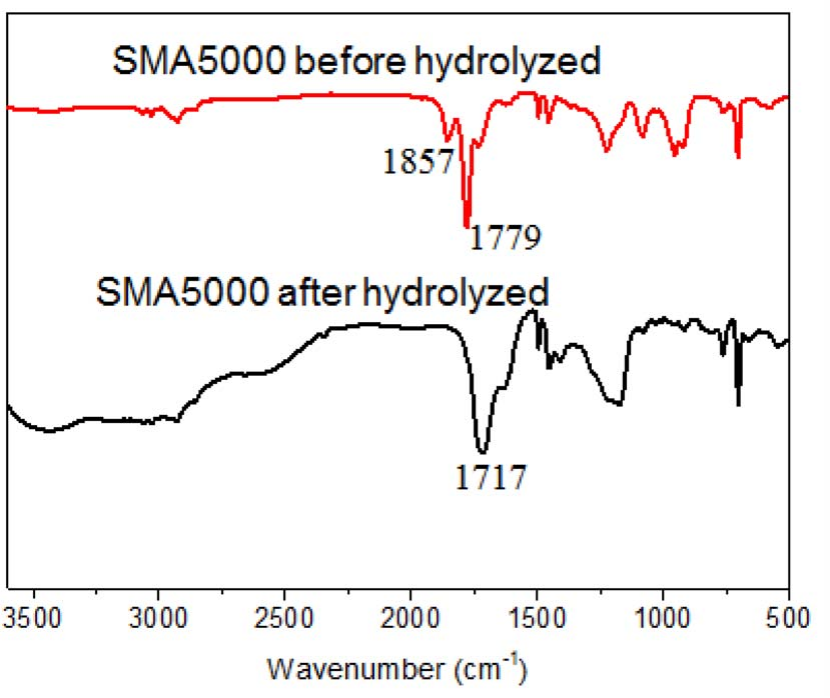
Infrared spectra of SMA5000 before and after hydrolyzed.

The water solubility and dispersion ability of SMA could be improved when the anhydride group of SMA was hydrolyzed to hydrophilic carboxylic acid under alkaline conditions. When SMA and the NaOH aqueous solution (equimolar dosage of anhydride) were mixed and reacted at 80 °C for 12 h, the light yellow transparent hydrolyzed SMA solution was obtained. The infrared spectra of SMA5000 before and after hydrolyzed were determined. The peaks at 1857 and 1779 cm^−1^ characteristic for carbonyl stretching bands from anhydride are observed (Fig. 4). After hydrolyzed, the characteristic peaks of carbonyl stretching bands from anhydride is disappeared, and ester peak corresponding to carboxylic acid (1717 cm^−1^) is observed (Fig. 4), which indicates that the anhydride had been completely hydrolyzed to carboxylic acid.

### 3.4 Effect of the *M*_n_ of SMA on the particle size of TiO_2_ slurry

TiO_2_ of Longmang R-996 is the rutile TiO_2_ which was prepared by sulfuric acid method, and the theoretical average particle size is 230 nm. The surface of R-996 is coated with silicon, aluminum and zirconium, and R-996 has good pigment properties, such as good whiteness, strong covering power. So R-996 is widely used in the coating industry. As we all know, the dispersant plays two roles in the dispersion process of TiO_2_. Firstly, the dispersant can reduce the surface tension between TiO_2_ and solution, and make TiO_2_ easy to be wetted and dispersed, then the particles size of dispersed TiO_2_ is close to the theoretical average particle size of original TiO_2_. Second, the dispersant can be adsorbed on the surface of TiO_2_ by electrostatic adsorption, van der Waals force and hydrophilic–hydrophobic interaction, so as to prevent particle reaccumulation through steric resistance or electrostatic repulsion.^33^

Table 2 shows the effect of different *M*_n_ of SMA on the particle size of TiO_2_ after diluted. As observed from Table 2, because of the small specific surface area of TiO_2_ particles, each SMA had good dispersibility to TiO_2_ in water. And the obtained particle size was close to the theoretical average particle size of original TiO_2_. However, the change of *M*_n_ of SMA has a certain effect on the particle size of pigment TiO_2_. The particle size of TiO_2_ dispersed by SMA3000 was 273 nm, and the particle size of TiO_2_ dispersed by SMA15800 was 339 nm. It was found that when the theoretical *M*_n_ of SMA was larger than 3000, the particle size of TiO_2_ increased with the increase of *M*_n_ of SMA. A possible reason might be that when the SMA was adsorbed on the surface of TiO_2_, it would form steric hindrance. The higher the *M*_n_ was, the greater effect of the steric hindrance was formed, so the particle size of dispersed TiO_2_ would increase with the increase of *M*_n_ of SMA. In addition, when the *M*_n_ of SMA was 15 800, the particle size of TiO_2_ increased obviously. It might be due to the long polymer chain of SMA forming many “bridges” between dispersed TiO_2_ in water and causing aggregation, which resulted in the increase of particle size of TiO_2_.^34^ However, as SMA1500 was used, the particle size of TiO_2_ was larger than SMA3000. It might be that the *M*_n_ of SMA1500 was too low to form steric hindrance effectively in the surface of the particles, which caused a small amount of particles to aggregate in the surface of TiO_2_. SZ40005 is the industrialized product of Polyscope company. Tested by SEC, the *M*_n_ of SZ40005 was 2800, and *Đ* was 1.60. The *M*_n_ of SZ40005 was relatively low, but it exhibited a bit of broadened *Đ*. When TiO_2_ was dispersed by SZ40005, the particle size of TiO_2_ was 349 nm and it was the largest one in the test range. The reason may be that SZ40005 was produced by a conventional free radical polymerization. Although the average *M*_n_ of SZ40005 is relatively low, there are a small number of macromolecular chains with huge molecular weight, which leads to aggregation caused by “bridges”.^35^ So the particle size of dispersed TiO_2_ increases when SZ40005 used.

**Table 2:** Effect of SMA on particle size of TiO_2_ in slurry

Entry	Sample	Size (nm)
1	SMA1500	288 ± 15.2
2	SMA3000	273 ± 11.3
3	SMA5000	286 ± 14.9
4	SMA8000	291 ± 13.4
5	SMA15800	339 ± 17.6
6	SZ40005	349 ± 16.4

### 3.5 Effect of the *M*_n_ of SMA on the viscosity of TiO_2_ slurry

Viscosity is an important index of the slurry. The viscosity of the slurry not only determines whether it is convenient to use, but also affects the energy consumption required for grinding. Last but most important, the viscosity affects the performance of the coating, such as whiteness, covering power and so on.

The results of the effect of SMA with different *M*_n_ and SZ 40005 on the viscosity of the slurry with 70% of TiO_2_ are shown in Table 3. According to Table 3, the viscosity of slurry with SMA5000 as dispersant is the lowest one and the paste viscosity of 70% of TiO_2_ is only 76.6 cp. However, with the *M*_n_ of SMA is lower or higher than 5000, the viscosity of TiO_2_ slurry dispersed by SMA mentioned above increases.

**Table 3:** Influence of SMA with different *M*_n_ on the viscosity of TiO_2_ slurry

Entry	Sample	Viscosity (cp)
1	SMA1500	87.3
2	SMA3000	84.6
3	SMA5000	76.6
4	SMA8000	98.0
5	SMA15800	254
6	SZ40005	157

While the *M*_n_ of SMA is 1500, the viscosity of slurry is 87.3 cp. It may be due to the shorter of the polymer chain, the smaller steric hindrance formed on the surface of the particles, which results in a slight increase in viscosity. When the *M*_n_ of SMA increases to 15 800, the polymer chains adsorbed on the particles surface would intertwine with each other, resulting in a notably increase of the viscosity of the slurry. As to the industrial SZ40005, although the average *M*_n_ is low, the polymer chains are heterogeneous. Therefore the presence of a small number of macromolecular chains with huge molecular weight significantly increases the viscosity of the slurry, and the viscosity is 157 cp. Thus the SMA5000 is the most efficient dispersant to TiO_2_ in aqueous solution among the SMA copolymers tested.

### 3.6 Effect of the *M*_n_ of SMA on the flow property of TiO_2_ slurry

**Fig. 5:**
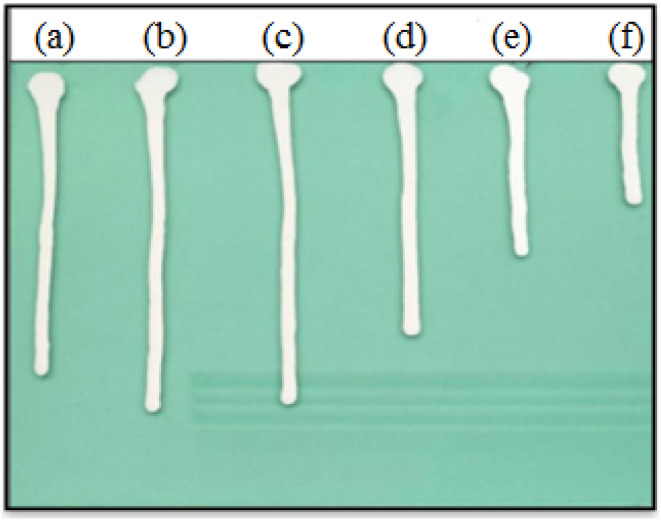
Optical photograph of the influence of SMA and SZ40005 on the fluidity of TiO_2_ slurry, (a) SMA1500, (b) SMA3000, (c) SMA5000, (d) SMA8000, (e) SMA15800, and (f) SZ40005.

The fluidity of the coating has a direct impact on the construction process of coating. So the flow property of TiO_2_ slurry dispersed by SMA with different *M*_n_ was carried out on an angular glass plate. Fig. 5 shows the flow property of TiO_2_ paste dispersed by SMA with different *M*_n_ and SZ 40005. It can be concluded from the graph that the effect of *M*_n_ of SMA obtained by RAFT polymerization on the flowability of TiO_2_ paste tends to change similarly with that of the viscosity of slurry, but there is a slight difference. Although there is a viscosity difference of 8 cp in the slurry dispersed by SMA3000 and SMA5000, the flow velocity on the glass plate is very close. The viscosity of the slurry dispersed by SMA5000 is smaller than that of SMA1500, and the flow velocity of the two has little difference. Interestingly enough, as to the SMA15800, the viscosity measured by rotary viscometer is much higher than that of SZ40005, but the flow velocity on the glass plate is faster. It indicates that the homogeneity of *M*_n_ has a great influence on the flowability of the slurry. Fig. 5 illustrates the *Đ* plays an important role on the dispersion of TiO_2_ in aqueous solution. When the *Đ* of SMA was lower than 1.20, the TiO_2_ could be absorbed by the polymer chains with uniform and small molecular weight (*Đ* < 8000), then the TiO_2_ could be stabilized by the “polymer shell” formed on the surface of TiO_2_. As a result, TiO_2_ slurry with excellent flow on the angular glass plate was obtained. When the *Đ* of SMA was higher than 1.50, a small number of polymer chains with huge molecular weight would lead to “bridges” between the TiO_2_, then some TiO_2_ particles aggregated together. Consequently, TiO_2_ slurry with poor flow on the angular glass plate was obtained. And Table 2 and Fig. 5 show that the smaller the particle size of TiO_2_ slurry was, the better performance of the flow velocity on the glass plate was. Overall, the dispersion effect of SMA based on RAFT technology is obviously better than SMA obtained by the free radical polymerization.

## 4 Conclusion

SMA copolymers with controllable *M*_n_ and narrower dispersity were prepared by RAFT polymerization, and the dispersing ability of SMA copolymers with different *M*_n_ and *Đ* for dispersion of TiO_2_ in aqueous solution was conducted in this study. The main findings are summarized as follows:

(1) The SMA with controllable *M*_n_ and narrower dispersity was prepared by RAFT polymerization using DDMAT as chain transfer agent, and the molar ratio of St to MAh was 1 : 1. The *M*_n_ of SMA measured by SEC was close to the theoretical *M*_n_, and the *Đ* is narrow (*Đ* < 1.20). The RAFT polymerization of SMA can be carried out under mild condition, and the conversion of monomer achieves to 99.1% after 24 h. So the RAFT polymerization of SMA is suitable for industrial production.

(2) By adjusting the molar ratio of monomer to DDMAT, the SMA copolymers with the *M*_n_ of 1500, 3000, 5000, 8000, and 15 800 were synthesized. The *Đ* of SMA copolymers were all lower than 1.20, and that of the industrial SZ40005 was 1.60. So the *Đ* of SMA obtained by RAFT polymerization is obviously narrower than that of SZ40005.

(3) SMA copolymers with different *M*_n_ were hydrolyzed under alkaline condition, and the dispersibility of hydrolyzed SMA copolymers for TiO_2_ in aqueous solution was studied. The results show that the *M*_n_ of SMA has a great influence on the performance of dispersant. When the *M*_n_ of SMA is between 1500 and 15 800, the performance of dispersity TiO_2_ in water by SMA5000 was the best. The viscosity of slurry with 70% of TiO_2_ is only 76.6 cp, and the slurry has the best fluidity than others.

(4) The *Đ* also has a greatly effect on the performance of SMA dispersant. The *M*_n_ of SZ40005 is 2800, and the *Đ* of SZ40005 is 1.60. With SZ40005 as dispersant, the viscosity of slurry is much higher than that of SMA3000 (*M*_n_ = 3000, *Đ* = 1.11), and the flow velocity on the glass plate is much slower than that of SMA3000. Interestingly, as to the SMA15800, the viscosity of slurry is much higher than that of SZ40005, but the flow velocity on the glass plate is faster, which means that the *Đ* plays a more important role on flow velocity than viscosity of slurry. Above all, the dispersibility of SZ40005 for TiO_2_ in aqueous solution is inferior to that of SMA prepared by RAFT polymerization.

The thesis analyzes the correlation between the *M*_n_ or *Đ* of SMA and their dispersion performance on TiO_2_ in aqueous solution, which can provides beneficial reference to synthesize high performance water-borne dispersant of SMA in industry.

## Ethical approval

This article does not contain any studies with human participants or animals performed by any of the authors.

## Conflicts of interest

We declare that we have no conflicts of interest to this work. We do not have any commercial or associative interest that represents a conflict of interest in connection with the work submitted.

## Supplementary Material
